# Dr. Bright's Reports of Medical Cases, Illustrating the Symptoms and Cure of Diseases

**Published:** 1832-04-01

**Authors:** 


### Medico-Chirurgical Review,

### No. XXXII.

JANUARY 1, to APRIL 1, 1832.

### I.

REPORTS OF MEDICAL CASES, SELECTED WITH A VIEW OF ILLUSTRATING THE SYMPTOMS OF DISEASES, BY A REFERENCE TO MORBID ANATOMY, &c. By *Richard Bright*, M.D., F.R.S. &c. Longman, 1831.

[Last Article.]

No other of the sciences is half so unprecise in the adjustment of its etymologies as medicine. Terms are employed in it now, terms have been employed in it for centuries, which have never been defined, or, if they have, possess significations so loose and unattached, that to all practical purposes they are unintelligible. Take *irritation* for an example. What has been meant by irritation—what is meant by it? If the various applications of this term be collated and compared, we defy any lexicographer in Christendom to construct a definition of it out of such materials as will bear upon one half of them. By some it means simple stimulation, whether of function or tissue; but no two of this party can agree in fixing upon the precise amount of increased activity, to which the irritated function or tissue is excited. With others it implies somewhat more than mere *excited* action—the first grade of *altered* action; but whether this change of action amount in tissue to change of structure, and in function to change of secretion, is not ascertained. By a third class we find irritation employed to express chronic, subacute, and even active inflammation, together with all the states and conditions which it is made to denote by the two preceding parties; but these unschooled grammarians are at a loss to draw the line of demarcation between an irritated state of organ, depending on altered structure, one arising from the first stage of inflammation, and another proceeding from over-active but still unaltered function, between all of which they feel that there ought to exist a difference, were it only for practical convenience. Dr. Bright has evidently experienced the annoyance resulting from this unlicensed and unmeaning use of the term irritation, and it is not to be wondered at, if he have, in a work exclusively devoted to diseases of the brain in which irritation and inflammation so often wear each other's mask, are so intermingled, or pass and repass into each other's state with so much facility, been occasionally betrayed into an application of this word, which custom has sanctioned because convenient, and criticism has tolerated although improper.

The section on which we now enter is appropriated to diseases of irritation, including hysteria, chorea, neuralgia, epilepsy, tetanus, and hydrophobia; "some of which, the Doctor admits, are strictly functional, while others occasionally owe their origin to structural changes." Hysteria and chorea are, perhaps, tolerably pure specimens of the effects of irritation, considering this word, as it ever should be considered, to denote a mere state of functional or textural excitement, with which no organic alterations nor any inflammatory action of consequence intermix. But neuralgia, tetanus, hydrophobia and epilepsy, not only so often arise from actual disease of texture, but are so often complicated with no ordinary indications of local inflammatory action, that in strict propriety of language they are no more pure diseases of irritation than delirium tremens is a pure disease of inflammation, although catalogued and described in these "Reports" among such. The action which they frequently portray "is," to use the language of the Doctor's *preface*, "irritation so mingled with inflammatory action as at least to deserve the name of inflammatory irritation." All these nervous affections, are, however, so closely allied to each other, that to have separated them and to have treated of them separately might have given rise to frequent repetition and inconvenience without rendering their essential characters more perspicuous, or their cure more practicable. The inflammatory action, with which they are often complicated, seldom admits of decisive depletion, and is not unfrequently aggravated by such treatment; and, although what was at first irritation, in the strictest meaning of the phrase, may at last terminate as well as be accompanied by organic disease, yet experience has shewn that the most successful practice—

—"Proceeds on the idea that irritability depends upon diminished tone, and is apt to be accompanied by congestion; and that a certain state of the vessels, either with or without organic disease, is often the irritating cause:—that, therefore, sanguineous depletion may be necessary either to relieve the brain from blood which is depressing the nervous power, and is in this way truly a source of diminished tone; or to take off such partial congestions as arise in the course of the disease; or to allay such local actions as may precede important organic changes, or may be afterwards induced by morbid structure: but that sanguineous depletion is to be used with caution, because it has, when unnecessarily employed, a direct tendency to diminish tone and increase irritability;—that purging, for nearly the same purposes as bleeding, is often useful; but is much more frequently admissible, as it tends to promote more healthy actions without diminishing power:—and that when obvious sources of irritation and obvious depressing causes are removed, it is to those remedies which more directly allay irritation, and to those which increase the tone, and thus diminish the irritability of the system, that we are to look with the greatest confidence; for which purposes the mineral preparations, as those of iron, zinc, arsenic, and silver, are powerfully efficacious, and may be occasionally administered with hyosciamus, combined with camphor, and some of the diffusible stimuli, which of themselves often act very favourably." 609.

*Hysteria.* There is certainly no organ in the female constitution, which maintains such frequent and intimate communion with distant textures and functions, as the uterus, whether in its pregnate or unpregnate state. The slightest derangement in its functions is instantly succeeded by a whole train of sympathies, as distinct as they are distant. Each organ acknowledges how seriously it is interested, each function proves how extensively it is implicated. Hence does it happen that there is no class of diseases, to which the female is exposed, so changeable in their symptoms, so capricious in their movements, and so fluctuating in their seat, as those in which this organ participates, and of these, perhaps, none is more uncertain and less uniform in every respect than hysteria. No clown, that ever enlivened a pantomime, has assumed a greater variety of forms, attitudes and features. It is a disease, which possesses to a marvellous extent the property of ubiquity. It sometimes attacks structures, and sometimes functions; now it wears the garb of spasm, then of palsy; in this instance it presents symptoms of inflammation, in that of extreme irritability; here it appears most prominently in organs situated at the extreme point of the circulation, there it concentrates itself exclusively in the region of the uterus. These various and opposite states of disease it, no doubt, seldom apes in an unmixed form. The inflammatory symptoms are usually more or less complicated by such as are purely nervous; its paralytic features are generally intermixed with indications of excessive action in some organ; and such instances, as are marked by mental disturbance, likewise betray more or less spasmodic ailment. In this way it is rendered tolerably evident that, however we may theorize upon this disease, its influence is principally exerted on the nervous system, so that it becomes a point of considerable practical importance to recognise it from purely inflammatory diseases on the one hand, and purely paralytic affections on the other. The inflammatory disease, which Dr. Bright has known hysteria most frequently to imitate, is peritonitis; and "nothing," he observes, "but the distinct history of the case and the careful watching of its progress can sometimes enable us to detect the true nature of the complaint"

"In general, some great incongruity of symptoms will be detected;—a tenderness of the abdomen, indicating inflammatory action, beyond any thing which the pulse, or the condition of the tongue, would authorize us to infer; a hurry and even labour of respiration more marked than in the embarrassed breathing of peritoneal inflammation; a sudden subsidence of symptoms, and their sudden return; a shifting and changing of the tender or painful part; and sometimes the decided intervention of hysteric symptoms, and the very frequent accompaniment of some evidence of mental causes, or of irritation and deranged function in the uterus itself. All these, together with the general aspect of the individual, will frequently be guides and indications to assist us." 453.

Not unfrequently it apes inflammation of the liver so closely, as to require considerable tact in detecting the fraud. In the following case both hepatitis and peritonitis were simulated; and, although the active treatment, which these supposed affections called forth, was succeeded by marked relief, the alleviation of symptoms which they occasioned was only temporary, and was ultimately followed by pernicious consequences.

(206.) The daughter of a medical man was supposed to labour under inflammation of the liver and peritoneum, in consequence of being frequently seized with pain and tenderness of the abdomen, more especially in the right side; for which she had been often bled to a large extent, and put under the influence of mercury. The attacks came on rapidly and disappeared as quickly; her pulse was quick, her nights were restless, and she was occasionally delirious. She was affected with dysmenorrhœa and leucorrhœa, and her lower extremities were so weak as to be almost paraplegic. From the frequent large bleedings her aspect had become blanched and puffy, and this debility arising from disease had been evidently increased by the treatment. Astringent lotions were ordered for the leucorrhœa, and zinc and gentian, persisted in for several weeks, completely dissipated every symptom.

In some instances the spleen, in others the kidneys, and in others the uterus are the suspected organs. The heart, lungs and pleura are also occasionally involved, and the brain and membranes do not always escape. In this way has it happened that hysteric patients, with weak and nervous constitutions, whose stamina were unequal to the onus of active remedies, have been reduced until they either sunk under the treatment, or gave way to an affection which, under more skilful management, might never of itself have been sufficient to extinguish life. In the 208th history, the Doctor records what he considers the symptoms of hysteric headache, which are so very similar to those of idiopathic derangement, that some may be found to question their nervous origin.

(208.) A young woman, whose catamenial discharge was somewhat irregular, became affected with at first slight, but afterwards excruciating headache, attended with intolerance of light, a frequent sharp pulse, furred tongue and vomiting. Leeches were applied to the temples, and twelve ozs. of blood were drawn from the arm; but the blood was uninflamed and the pain undiminished. Free purging was then tried, but with little benefit. A blister was applied to the neck with more advantage, and the compound steel mixture wholly removed these symptoms.

Not only is hysteria equivocal in the organs it apparently attacks, but it is equally so in the kind of action which it seemingly establishes. The functions, as well as the structure of organs, very distantly situated from the parts originally diseased, are disordered, giving rise to a host of most discordant and anomalous phenomena. Thus the respiratory apparatus is very frequently implicated, rendering the respiration violent and irregular—the organs of deglutition are often deranged, producing difficult or impracticable swallowing—the vocal apparatus is invaded, causing the voice to be either wholly suppressed or emitted by spasmodic ejaculations—the heart is attacked, and the consequences are violent palpitations and irregularity in the pulse—the functions of the brain are confused, and melancholy, delirium or madness follows—the entire muscular system is enspasmed, and partial or general convulsions result. These are the most frequent and favorite simulations of hysteria, and very seldom does any pure instance of this affection happen, which is not marked by more or fewer of these spasmodic symptoms; but the reasons for one class of functions suffering in one case, and a different class laboring in another, are only to be found in some idiosyncrasy of constitution, or susceptibility of structure.

A lady lay for more than a fortnight, suffering incessantly from a convulsive effort resembling hiccup, which was distinctly audible in different remote parts of the house, and which had so seriously reduced her strength, that the result was looked forward to with anxiety. But a blister to the nape, cold to the head, and frequent doses of æther and brisk purgatives, so rapidly dissipated this unfavourable state, that in two days she was quite well. A female who had diseased mamma, was seized with alarming dyspnœa, attended with a sense of tightness across her chest, which some considered to depend upon inflammation of the lungs. The pulse was quick, but weak; the respiration was laborious, but unattended with cough; her feet were cold, and she was in a state which might have resulted from sudden effusion into the chest, or the bursting of an aneurism. This was hysteria, and assafœtida was its cure. A woman was supposed to suffer from constriction of the œsophagus, in consequence of the difficulty and pain with which deglutition was performed. But, when a probang was introduced, to discover the seat and size of the contracted part, the irritation of the instrument induced an hysteric paroxysm, thus betraying the true cause of the dysphagia; and, what is somewhat peculiar, the fit, which had been thus provoked, was immediately followed by hysteria in *several* females in the ward.

The instances, which we have now on record of this disease propagating itself by sympathy, are so numerous and so well authenticated, that this communicative property must be regarded as an occasional feature of hysteria. Some, anxious to explain such cases in a more intelligible and ordinary way, have endeavoured to account for them upon some epidemic cause, or some local peculiarity. But no solution of this nature, yet seen by us, has been a whit more intelligible than the one it attempts to supersede. We yawn by symphathy, we laugh by sympathy, we weep by sympathy, we stammer and lisp by sympathy, and are not such sympathetic acts as unintelligible, although indisputable, as any other form of convulsive movements? Besides, in the great majority of those instances in which hysteria, chorea, or convulsions have seemed to spread by sympathy, no local cause has been discovered, which could be etiologically connected with the phenomenon.

A middle-aged woman came into hospital with loss of voice and speech, her tongue was protruded in a straight line from her mouth, and the lower jaw shivered when she attempted to speak, as from ague. She had been subject to hysteria, and for the last four years felt occasional numbness and pricking sensations in her hands. Some of these symptoms appeared indicative of palsy, but more minute inquiry convinced the Doctor that they were hysterical. She lost fourteen ounces of blood from the neck by cupping, her bowels were actively moved, and she took camphor mixture with tincture of assafœtida. Relief immediately followed, and in a few days she was quite well.

It is not uncommon to meet with hysterical females, who suffer under great weakness, if not total palsy of the lower extremities. On the most minute examination of the spine, neither tenderness nor deformity is discoverable. They have sustained no known injury in either the head or back, and, although unable to walk or stand, the urine and stools are passed regularly, and with complete cognizance of the will. If the catamenial discharge be examined, it will in general be discovered faulty, either in its periods of return or quantity, and local stimulants with moderate purging and cordial tonics are found to be the most beneficial remedies.

(218.) A delicate young woman was placed under the Doctor's care, having lost the use of her lower extremities for the last seven years. The spine was carefully examined, but nothing unnatural could be detected. Her catamenial secretion had been obstructed about the period when paraplegic symptoms occurred, and for the last two years had wholly disappeared. Her bowels were constipated, and she occasionally complained of headache. The condition of the stomach and bowels was improved by tonics and antimonial ointment, and blisters were applied to the loins. For a considerable time little improvement was produced, but the catamenia gradually returned, and after having been seven or eight months under treatment, she got well and "left the hospital walking, as if she had never been ill."

In constitutions of the hysteric temperament, not only is the entire nervous system in an acutely irritable state, but the mental faculties frequently present great peculiarities. The imagination is vivid and most fanciful, the taste is eccentric and most capricious, the perception is active and most deceptive, the judgment is weak and undetermined, the passions are feelingly alive to the slightest stimuli. The degrees, in which these mental peculiarities exist, are as numerous as the constitutions which they inhabit, and as varied as they are fugitive. Sometimes they deviate so slightly from the ordinary standard, as scarcely to betray any marked difference ; but it occasionally happens that the mind is so warped by the bodily infirmity, as to be most seriously unhinged. A thousand complaints are fancied by the hysteric so circumstanced, which are not the less injurious because they are imaginary. Being beyond the reach of detection, they are of course beyond the influence of remedy. Females have been thus confined to bed for years, their strength has been slowly undermined by confinement and mental agony, and although no skill, which could be consulted, nor any diligence which was employed, could discover any actual disease, they have at length expired with all the indications of a most diseased and dilapidated fabric. It sometimes happens that, after medicine has done its utmost in such cases without effect, the constitution suddenly and unaccountably re-adjusts itself without assistance, as in the following instance.

(219.) A young lady of amiable dispositions and cultivated mind had been confined to bed for above nine months, before the Doctor was called in. At that time every attempt at motion was succeeded by a paroxysm of agony and agitation, which almost overcame her. She had no appearance of important disease, and the range of medicines, which had been tried for her relief, but to no purpose, was so extensive, that little hope could emanate from that quarter. She complained of constant throbbing in her temples, and she had almost lost the use of her lower extremities. As some soothing effect seemed to be produced by stimulating enemata and pills which she had been taking, they were exchanged for bread-pills and injections of plain water, on the belief that her complaints were principally imaginary, and these inert medicaments were found of equal efficacy. After this time she was frequently visited by the author, but no improvement was perceptible, until "once, after having attended altogether about nine months, I called after an absence of nearly a month ; her sister met me at the street-door with a smiling face, to tell me that our patient was quite well : and on inquiry she related how three mornings before, under a deep religious impression, she had completely recovered all her powers; and I found her sitting up working and amusing herself, as if she were completely convalescent from some ordinary illness."

Were Irving's disciples to meet with a few such cases as that of this young lady, the story of Miss Fancourt would be noised abroad once more with redoubled confidence, and the gift of healing would be only equalled by the gift of prophecy! In this instance there was obviously no more serious malady than functional disturbance, and although no allusion is made to the state of the catamenial dischage, there is little doubt but that it had been deranged, if not wholly arrested. The hopeless condition, to which this hysterical hypochondriasis may reduce the constitution, is well illustrated in the succeeding passage.

"I am at this time occasionally consulted about a lady, who has been confined to her bed for between four and five years, giving way to all the morbid feelings of this dreadful disease to such a degree, that, although no one, of the many who have been consulted, has ever been able to detect any actual disease, she is now emaciated to the last degree; her stomach, by long yielding to its apparent debility, is at length reduced to such a condition that it will admit of scarcely any nourishment, and the patient exists almost entirely on brandy and water, of which she drinks a most incredible quantity. She sits propped up in bed, speaking in a whisper: her countenance blanched; her hands emaciated; her eyes closed to the light, and dreading almost a footstep in the chamber, on account of the pain it excites in her head.—In her case, remedies are but imperfectly persisted in, and the disease fostered by the ill-judged sympathy of over-fond relations." 463.

No doubt the uterine derangement which usually accompanies this disorder, whatever symptoms it may assume, may and does not unfrequently produce actual disease in both textures and functions previously susceptible, and no organ seems more obnoxious to these sympathetic affections than the brain. Hence the importance of obviating as much as possible the primary symptoms, and of attending to the manner in which the uterine functions are discharged. It is well known that the uterus may itself become so diseased by long irritation and abuse, as to convert what was only a periodical and purely functional derangement, into a permanent and incurable disease. In the following history such symptoms supervened upon a diseased condition of the cervix uteri, as set treatment at defiance, and exhausted what had been a healthy and robust woman.

(220.) A female, upwards of 70, became the subject of violent nymphomania, attended with such mental disturbance as required restraint. This disease preyed upon her for nearly five years, and after reducing her from a state of robust and active health into a paralytic state of body and imbecile condition of mind, it ultimately destroyed her. The head unfortunately was not examined, but the only disease of note to be found in either of the other cavities was situated in the uterus. At the orifice of this body grew a tumour of a vesicular character, as large as a hazel-nut, and along its internal surface were several smaller ones, which had formed beneath the mucous lining, but by enlarging had gradually pushed it before them, so as to appear within the uterine cavity. The cervix uteri was much thickened and indurated, and on being cut through two or three cysts of the size of peas were discovered in its substance.

The first organ, therefore, to which the practitioner's attention should be directed in hysteria, is the uterus. Should it prove either functionally or structurally deranged, such measures as are best adapted to remove these states must be employed, and any phenomena, which may arise from the sympathies of distant organs, must be treated as either originating in this source, or most intimately connected with it. As has been shown in several of the preceding cases, these sympathies are occasionally so severe, as to require distinct treatment. Local congestions form in consequence of local susceptibilities to action, and hence does it happen that local and even general bleeding must sometimes be resorted to. The Doctor, however, very properly objects to free depletion in all cases, where fugitive and anomalous pains arise; believing that tonic medicines will not unfrequently dispel what at first sight might appear the results of inordinate activity.

"I have seen pains the most acute, particularly in the head, only aggravated by the loss of blood, and yield readily to the compound steel mixture.—Cold applied to the head, and more especially the sudden application of cold, assists in diminishing the cerebral congestion : and by the shock of cold water to the surface of the face, it is not improbable that some direct effect is produced on the par vagum and its associated nerves, which seem deeply implicated in hysteric attacks.—Blisters are likewise very useful, applied over the parts affected with hysteric pains, and to the nape of the neck in various and frequent forms of such diseases.—In all the forms of hysteria, great assistance is occasionally derived from the class of remedies included under the general appellation of diffusible stimuli; as the ammonia, the camphor, and the æthers ; and to the former of these I have seen many obstinate cases decidedly yield ; and amongst others, severe and long-continued local pains, in the cure of which the belladonna plaster has also appeared materially to assist." 467.

*Chorea.* A disease in some respects resembling hysteria is St. Vitus's dance ; but as in hysteria the muscles of involuntary motion are most affected, in chorea those under the direction of the will are principally concerned. In many cases it has obvious connexion with uterine derangement; but in others either the sex or age of the patient precludes the possibility of such relationship. Like hysteria it may arise from sympathy ; it is also certain that it has originated in fright ; injuries of the head have acted as its exciting cause ; and it has been so often found associated with rheumatism and dentition, that these diseases the Doctor apprehends may in some instances have favoured its appearance. It exists in an acute and chronic form ; varieties which are in many respects worthy of distinction. When it approaches with acute symptoms children are its most frequent subjects, and males are less exposed to it than females. The convulsive movements are violent, and if restrained wear out the patient, who sinks exhausted with dry brown tongue, muttering delirium, and other symptoms of advanced typhus. In the chronic variety these convulsions are less violent, seem to attack males more than females, are often continued by habit, proceed without danger, and are seldom cured. Chorea has occasionally lapsed into fatuity or confirmed madness; its analogy to epilepsy and tetanus is striking, and from the nature of the treatment which is most successfully pursued, the author is inclined to suspect a close relationship between it and delirium tremens on the one hand, and hysteria on the other. Dr. Bright has known it to terminate fatally in six instances. In two of these cases the patients were plethoric women, of an age most favourable to uterine irritation. In a third the woman was four months advanced in pregnancy; and in the fourth, which is fully recorded by the author, dissection disclosed abundant evidence of uterine disease.

There seems to be a predisposition in some families to chorea so strong and so well-marked, as to give to it in the eyes of some the appearance of an hereditary malady; but our knowledge upon this point is as yet too li-limited to entitle us to regard such occurrences as any thing more certain than accidents. The Doctor has attended a brother and sister in this disease, whose mother had been addicted to it when a child; and on another occasion, after having dismissed a little girl cured, her cousin, who had been living at a distance, and who had never communicated with her, took ill. The following cases will illustrate chorea, as arising from fright, sympathy, exposure to cold, irregular menstruation and diseased uterus.

(228.) A girl, aged 18, was affected with this disease, but the convulsions were principally confined to the left side. Four years before the present fit, she had an attack brought on by a fright received from a woman appearing to her in a white sheet, which continued for six months, and five months before admission into hospital she had been again alarmed by thieves breaking into her house, in consequence of which, as it was believed, the disease recurred.

The immediate effect of fright in this case was to derange the menstrual discharge, and it is not improbable but that in most such cases it is through this channel that the nervous system is disturbed. In the case immediately preceding the patient had also been frightened by having been shut up in a coal-hole, and was attacked by the disease ten days thereafter. But as she was only five years old, the menstrual functions could not have been influenced.

(226.) A girl of slender make was subject to chorea in a slight form for six months before admission into Guy's. She attended a school, in which a little girl was affected with the same disease, and no other cause could be discovered for her illness than that she either unconsciously or intentionally imitated this girl, and thus formed a habit which she could not afterwards shake off.

In the 233d history the disease wears a complicated aspect, being closely combined with rheumatism. The right leg and arm were first affected with severe pains from exposure to wet, and in about three weeks subsequently these rheumatic pains were displaced by choreal symptoms, which infested the same parts.

(237.) When the menses first appeared, Ruth Grenier first complained of numbness of the right arm, and irregular motion of the fingers of the right hand. The pil. aloes c. myrrha were given every second night, the mist. ferr. comp. every six hours, and a shower-bath was ordered every alternate morning. In the course of four days the catamenia appeared, but the choreal symptoms continued unabated. To relieve some sickness and headach, which had supervened, twelve leeches were applied to her temples, and a rose-draught was given; and ultimately the sulphate of zinc was substituted for the aloes, myrrh and iron, under the exhibition of which she got well.

It will be seen by this as well as several other cases that one side of the body is much more severely attacked than the other, and it sometimes occurs that the disease is so exclusively confined to one side, and so severe as not unfrequently to be mistaken for hemiplegia at the first visit. The next case proved fatal.

(239.) A girl, 13 years of age, six months before her admission into hospital, had an attack of rheumatism, from which she completely recovered; some time after which she had an ulcerated sore throat, attended with headache, globus hystericus and some mental delusion, and as these symptoms disappeared choreal spasms succeeded, her spirits sunk and her strength failed. The catamenia had not yet appeared, the bowels were habitually obstinate, the appetite voracious, the tongue white, and the pulse was 120. A blister was applied to the nape, a purgative of rhubarb and calomel was given, and the mist. ferri comp. was ordered at first in half, and afterwards in oz.-doses, six times a day. Her symptoms improved slightly under these medicines, but the pulse still kept up although languid, and objects appeared double. The pil. aloes c. myrrh. and extract hyosc. with camphor were added to the tonic mixture and continued for a fortnight, when they were changed for a pill composed of zinc and gentian, and to these afterwards was joined a small quantity of port. The disease, however, pertinaciously advanced, until cold affusion was had recourse to, by means of which some tranquillity was procured at night, and by increasing the dose of the zinc to ten grains every eight hours, and persevering in the use of stimulating purgatives and the shower-bath, in two months "she left the hospital well." After remaining "quite well" for many months she relapsed in consequence of some mental agitation, to which she had been exposed; but she again recovered, and being again exposed to some excitement in a more severe form, she again took ill, and died with the most confirmed and intense symptoms of chorea. The skull-cap was thin, there was a small quantity of water beneath the membranes, the surface of the convolutions was rather more vascular than natural, and the cerebral substance displayed rather too many vessels. The plexus choroides were turgid with blood, and the vessels running over the corpora striata and thalami were full and large, but there was no effusion within the ventricles. The pia matral covering of the spinal cord was rather vascular; between the cauda equina and half-way up the spine were seen five or six bony plates above the tenth of an inch in diameter, attached to the pia mater, but the corpora pyramidalia and olivaria were most carefully examined without betraying any marks of derangement. The uterus was large, and its cavity extensive. The right ovary contained a cyst filled with a tenacious red substance, and the right fallopian tube, which was quite pervious, had its fimbriated extremities tipped with botryoidal-shaped deposits of semi-transparent bone. Attached to the ligaments of the uterus on each side was a small vesicle hanging by a peduncle, along which vessels were seen to pass, and a few vesicular bodies were found in the left ovary.

The extent to which uterine derangement may influence the general health is here well illustrated. How far some of the morbid products disclosed by dissection were cause or effect, it may be difficult to decide; but it appears more than probable that the disappointment in love, and other mental disturbances which this girl suffered, had more than a general effect either in predisposing to or advancing the uterine disease. The bony deposits upon the spinal cord, whether formed late or early in the disease, must have excited irritation ; but it is likely that the cerebral congestion was only one of its effects.

When the patient is young and plethoric, or in a pregnant state, the removal of a little blood from the arm may be beneficial; but as a general rule the Doctor disapproves of sanguineous depletion in this disease, except in small quantity. The application of leeches or glasses to the temples, nape or spine is not unfrequently required ; but even local depletion must be regarded more as a preventive of dangerous congestions, than as calculated to remove the specific symptoms. Where the chorea is uncomplicated with rheumatism, or any other more purely inflammatory affection, leeches and the lancet will be better borne and more required ; but the experience of our author is much more favourable to tonic medicines, accompanied by moderate purging. The compound scammony powder or calomel and scammony he recommends for children, aloes and myrrh for patients of more advanced age. He has sometimes given calomel in five-grain doses for three or four nights in succession, following it up with a purgative each morning; and in obstinate cases he has applied issues, setons and blisters to the nape ; but—

"Tonic remedies are those on which the chief reliance is to be placed, though they always require to be combined with purgatives. It will often be necessary to persist in the use of tonics for a long time, gradually increasing the dose. The mineral tonics are most efficacious ; and of these the sulphate of zinc cautiously increased, and the subcarbonate of iron, are least liable to do harm.—I generally begin with a single grain of the sulphate of zinc, and increase it every second or third day, one grain at a time ; in which way a child of ten years of age, will often take ten or twelve grains in the form of pills, three times a-day, without suffering the least inconvenience. The zinc has sometimes proved effectual when the iron has not ; and, on the contrary, the subcarbonate of iron will sometimes succeed when the zinc has failed ; but it often requires to be increased to such an extent, that the powder becomes very disgusting to the patient, simply from its bulk. I have given it in doses of half an ounce and I believe larger doses have been given. In some cases, more particularly those which are accompanied with amenorrhœa, the compound steel mixture, repeated as frequently as every fourth hour, is an efficacious mode of administering iron, and is less unpleasant than the powder of the subcarbonate. I have occasionally given with advantage the tincture of the muriate of iron. I have known instances in which arsenic has been successfully administered ; but I object to this remedy, where others less hazardous will act as well. On the same ground I should object to the use of the nitrate of silver, which has often permanently changed the colour of the skin, though I have seen it administered by others with utility in chorea; the sulphate of copper is another remedy, which I think objectionable on account of the irritation it often produces in the stomach." 471.

The 231st case is a good illustration of the effects of this practice. C. L. aged 16, of slender form, who had never menstruated, was affected with well-marked and rather severe symptoms of chorea, which some ascribed to fright. Colocynth and calomel, aloes and myrrh, and two-grain doses of the sulphate of zinc every eight hours were the medicines at first employed, and when she came under the author's care she was entirely confined to bed in a constant state of convulsive action during the day, throwing herself from one side of the bed to the other, unable to articulate, and swallowing with the greatest difficulty." The zinc was increased to five grains every six hours, five grains of calomel were given at night, and castor oil in the morning. Still, however, the disease advanced, and Griffith's tonic mixture was substituted in ounce-doses every two hours for the zinc, five grains of the hyosciamus extract were ordered as a night-pill, and ten ozs. of port were given. In less than a week her improvement was quite visible, and in about a month she was "quite free from all symptoms." This patient had a recurrence of her disease five months after, but she was again cured by the same remedies.

The cold shower-bath adds much to the efficacy of internal tonics, and may be employed on alternate mornings with the purgative medicines. The spasmodic movements are sometimes so severe, however, as to render its application difficult, when cold affusion may be substituted with advantage. In the fatal case, which has been extracted, its good effects were obvious and considerable, and the 229th history affords another instance of its value. Where there is neither fever nor inflammatory symptom the diet should be generous, and even a little wine prudently administered has been followed by marked improvement in cases of a very unpromising description. Anodynes likewise tend to allay the excessive irritability by which this disease is characterized and hyosciamus joined to camphor is the preparation, which the Doctor has found most serviceable for this purpose. Under the most judicious management, however, chorea is a disease which can seldom be prevented from re-appearing in some degree at some future period of life, although banished for a time. The mobile and susceptible state of the nervous system, on which it either depends, or to which it gives origin, leaves the patient in great danger of relapse, and no preventive plan yet devised has been found equal, either in this affection or that last described—hysteria—to counteract this predisposition. In several cases detailed in the work before us the patient had either suffered from the disease before coming under the Doctor's care, or was subject to it afterwards ; and in the 224th history the child was treated for the same symptoms at three different periods, having suffered an attack each year for three successive seasons.

*Neuralgia* appears in various forms, and attacks different parts. In one case it appears as sciatica, and apes with close resemblance the character of acute rheumatism. In another it wears the symptoms of tic douloureux, and presents a less inflammatory, but much more painful character. In a third it appears as an intermittent headache, attacking however only one-half of the head, and therefore denominated hemicrania ; and in a fourth it has been seen to simulate angina pectoris, cardialgia and asthma. That the nervous system is the seat of this distemper is most undeniable, and that the nerves affected are either inflamed or highly irritated is evident from the pain experienced along the track of their position, when pressure or any other external irritant is applied—sciatica has been by many regarded rather of a rheumatic than neuralgic nature, notwithstanding ; but, however frequently these affections may be associated with, or resemble each other, they certainly can not only exist apart, but the one is often little benefited by the remedies most commonly and most usefully employed in combating the other. Cupping along the seat of the sciatic nerve, followed by a belladonna plaster, and aided by a pill composed of opium, tartar-emetic and calomel, given every six hours and worked off by an occasional aperient, is the treatment recommended in the present instance. Colchicum, which is so beneficial in rheumatism, does not seem to exert much influence upon it; neither do the chalybeates, which have proved so successful in other forms of neuralgia: yet after the measures above proposed have dissipated the phlogistic symptoms, and have reduced the disease to a chronic state, the rust of iron has been advantageously employed.

In tic douloureux there are fewer indications of increased action, but much more intense pain. According to Sir H. Halford osseous disease is very generally the irritating cause; but the cases are innumerable, in which tic can be traced to other sources of excitement. Were spiculæ of bone, or osseous irregularities the general exciting cause, how does the President account for the present very successful treatment of this affection with the rust of iron? This substance will scarcely be supposed capable of shaving off either irregular growths, or carious sphincters, yet in a few days, nay in as few hours, has this medicine been found capable of dissipating attacks, which have endured for weeks! And how does it happen that the neuralgic symptoms are not as constant as the exciting cause? If the nervous irritation originate in osseous disease, the irritation should be as unintermittent as the irritating agent; whereas it is highly periodical in its continuance, and is most generally more or less connected with chylopoietic derangement. Indeed, we think it has been tolerably well proven, that this form of neuralgia, at least, is more a disease of function than of structure, and it is to this happy circumstance that we are to ascribe so much of its manageableness under tonic and alterative remedies. "It will, perhaps, be found," in the Doctor's opinion, "that as much depends on the character of the nerves irritated, as on the nature of the irritating causes, the irritation of course acting in such cases upon nerves of sensation, more than on nerves of voluntary or involuntary motion." In the following case, at least, the President's theory is illustrated.

(249.) A middle aged woman, who was thin and whose countenance was strongly marked by the effects of long suffering, was admitted into hospital for an extremely acute pain on the left side of her face, which *was seldom completely removed*, but became more severe in paroxysms. Every remedy, which had been tried, proved unavailing and she died. There was more than the usual quantity of water in the ventricles and upon the surface of the brain, which was rather soft and presented a slight mottled appearance in its medullary matter. The dura mater, immediately under the anterior part of the left middle lobe, was elevated by fungoid tumours, which collectively were equal in size to a pigeon's egg. The surface of the lobe corresponding with this tumour was depressed, softened and slightly adhered to the elevated portion of the dura mater. The bone beneath the tumour was carious; the mucous lining of the left nasal cavities was diseased; there was a soft pedunculated polypus of the shape and size of a raisin attached between the turbinated bones; but that part of the portio dura, which had been laid bare in the removal of the diseased parts, exhibited no marked appearance. With the exception of old pleural adhesions, and a tuberculo-hepatized state of the right lung, there was no disease of any consequence in any other part of the body. The *continuous* character of the neuralgic pains in this instance, considered in relation to the nature of the exciting cause, will have some influence upon a remark on this subject already made.

Mercury, either internally or externally employed with opium, so as to produce salivation, has more than once cured this disease; large doses of bark, repeated every hour, have afforded great relief; and the external application of tar to the parts pained has sometimes been successful; but the remedies most to be depended on are rust of iron and the arsenical solution given in large and frequent doses, at the same time preventing the bowels from falling into an unnatural state. Mr. Hutchison's merit, in drawing the attention of the faculty to the value of the subcarbonate of iron in the treatment of neuralgia, has now been very generally admitted; and, were it not for the grossly open and extensive plagiarisms which his work on this subject betrays, we could cordially acknowledge it in its fullest extent. But notwithstanding that it seems hitherto to have escaped detection, it should not be concealed that entire pages of the volume here alluded to are taken *verbatim et literatim*—sentiment and language, from an author—Fothergill—much Mr. Hutchison's superior in reputation, but who should not have been thus pillaged even for *the sake of science* because he was no longer among us to protect his property. Mr. Hutchison was, no doubt, quite capable of writing his own sentiments, as well as his own language; but since he either had not time, or would not take the trouble of doing so, both equity and honor claimed an acknowledgement of the fountain out of which he drew.

Division of the diseased nerve has been advised and practised, but whether from the nerve having been imperfectly divided, having afterwards united, or having been irritated by a cause which lay beyond the operator's reach, the knife has seldom effected a complete and lasting cure. If chronic disease of the neurilema, or substance of the nerve itself, be the cause of the neuralgic symptoms, unless the division of the diseased nerve take place between the diseased portion and the sensorium, the operation appears to us to be wholly useless. For, if a part of the diseased nerve be still left in communication with the brain, it matters little how much of it may have been removed by the operation, morbid impressions will still continue to be transmitted to the sensorium, and the original disease will still remain. As it is, therefore, frequently a mere point of conjecture, whether the excited nerve be irritated from sympathy or by an idiopathic cause, and as it is still more frequently conjectural to what extent the disease obtains, when it is the texture of the nerve itself which is implicated, it is too evident that an operation, so vaguely indicated, can seldom be productive of permanent good.

Hemicrania is so often connected with hysteric habits, that by some it has been supposed to be as nearly allied to hysteria as neuralgia, and there is no doubt but that it is much more obedient to medicine than any other form of this painful and obstinate disease. It is not unfrequent during lactation, and pregnancy seems favourable to its approach. It seldom wears a continued form, and its periodicity has suggested some similarity of character between it and genuine intermittent. However that may be, it is certain that bark, iron, Fowler's solution of arsenic, and such other remedies as are most successful in the treatment of ague, are equally efficacious here.

(251.) A short stout young woman was attacked with inflammation of the right eye, for which she was cupped and leeched, and when it was getting better she became suddenly seized with a severe pain, which shot from the right orbit across to the occiput of the same side, for which cupping, leeching and much medicine had been inffectually employed. When first seen by the Doctor this hemicranial pain was attended by a throbbing sensation anteriorly, and had an exquisitely tender circumscribed spot about the size of half a crown, at each extremity of the affected part. The pupils were dilated, but the pulse, tongue and skin were natural; the bowels were open, but the catamenia were irregular. Five drops of Fowler's solution were given in cascarilla infusion three times daily, and a pill composed of mercury, colocynth and galbanum was ordered every alternate night. For nine days thereafter no improvement occurred; indeed her headache was rather worse, and it was complicated with vomiting, numbness of the limbs and giddiness: but at this period a gradual diminution began to take place in her suffering, and in less than a fortnight she left the hospital cured.

Mr. Teale, in his interesting little work on neuralgia, which we favourably noticed at another time, has described a great many varieties of this disease, in addition to those noticed by the Doctor, which close examination discovered the cause of in a tender state of the vertebral column, or spinal cord. Anomalous pain about the scrobiculus cordis, along the short ribs, over the region of the spleen and liver, and even in the situation of the heart, closely simulating angina pectoris, have been carefully and in our estimation clearly traced to some inflammatory derangement either in the vertebral column, or its contents. These he found were generally removable by the application of leeches and antiphlogistic remedies, local and general. Whether our author have met with any forms of this disease depending on the same cause, he does not say, but in a field of observation so extensive as that which he enjoys one might presume that such cases were not unlikely to occur, and it were desirable to learn how far his experience corresponds with Teale's views.

After herpes zoster it sometimes happens, that acute neuralgic pains are experienced in the integuments formerly diseased; and unless the practitioner examine with some care the entire history of the case, he may be inadvertently betrayed into a very erroneous conception of its real character. In the 247th history, the patient's complaint had been mistaken "by an experienced practitioner" for "an internal malignant disease," for which a seton had been inserted into the left side of the chest. In the course of some weeks, however, the pains gradually yielded to opiates. In the 248th case the herpetic eruption died away in its usual course, but the pains which remained were severe, and quite unappeasable by opiates. A scruple of the subcarbonate of iron was ordered three times daily, increasing the dose as the cure proceeded; but—

"The relief was so rapid, that a very few days served to remove the pain entirely.

This is the only case in which I have employed this remedy, and I therefore will not lay great stress upon it; but if the utility of this mode of treatment should be confirmed by future experience, it will not only enable us to relieve a painful disease some days or weeks before it would probably subside of its own accord, but the remedy also serves to throw some light on the neuralgic nature of the pain, and the peculiar connection of the original disease with some affection of the nerves." 503

*Epilepsy* has sometimes been seen at the earliest age, as well as at the decline of life, but the most frequent periods of its occurrence are between seven and eight during the second dentition, and from fourteen to sixteen at the age of puberty. These two periods are equally critical and severe upon the constitution. Every weak and unprotected organ is tried to the utmost. Every function is put upon the test. Diseases, which had hitherto lain hid and undeveloped in their own seminal rudiments, are at those periods of trial called forth into activity; and predispositions, which before had been of themselves unable to awake those morbid principles which had been quietly awaiting their proper birth-time, are then fostered into operation.

The hereditary nature of epilepsy is now no longer a question. It can be traced with ease from parent to child, and it is by no means clear that, like hysteria and chorea, it may not be propagated by sympathy. Sometimes, but rarely, it occurs that one well-formed paroxysm is the only manifestation which it makes during an entire life; more frequently many years of interval separate one paroxysm from another; but generally its periods of re-appearance are short and uncertain. It has been known by the Doctor to recur no fewer than thirty times in the short period of twenty-four hours. Its period of return is sometimes quite fixed, but usually it is most irregular. In one instance it approaches during day, while the night is much more favourable for its approach in general. This capriciousness can sometimes be accounted for, by referring to the nature of the exciting cause. If epilepsy appear monthly in a female, the uterine functions may be fairly considered to have some connexion with its lunar periodicity; if it occur at night in preference to day, the cerebral congestion which attends a state of sleep may be recognised as a reason; and if it appear at unmeasured and uncertain intervals, some casual excess or accidental irregularity may be often found to account for it. There is more difficulty in discovering the cause of the multifarious modes, by which it approaches, and of the different phenomena, by which it is attended. In one patient the paroxysm is introduced by a long and intelligible train of precursory symptoms, while in another the fit occurs instantaneously, without the faintest premonition. Some are depressed by melancholy, while others are unusually cheerful before the occurrence of the fit. After a momentary absence of mind it occurs, without noise, convulsion, or any more outward sign than a vacant and inexpressive stare. Sometimes this fugitive mental annihilation, or rather absence, is complicated with partial convulsions,affecting more severely the face and hands. And in other instances the whole body is as violently excited as the mental functions are palsied and inactive. In the 252d case the symptoms wore the mildest form.

A large robust man, aged 36, with an ample forehead, was seized while at work with numbness of the left hand, which, passing up the arm, affected the left cheek, and the left half of the tongue to such a degree as greatly to impede his speech. This first attack continued for about twenty minutes, and on going off left some headache behind, which however did not prevent him from pursuing his work. Six weeks afterwards a similar attack occurred, this was followed by several others, and between the paroxysms he latterly felt so weak and nervous, that for seven weeks before he was placed under the Doctor's care, he had been obliged to give up his ordinary business. The feet and legs were ultimately affected as well as the hands and arms, but beyond transient vertigo the mind never betrayed any indication of disturbance.

The 264th history is a good illustration of epilepsy in the next, or second grade of severity. A boy, aged 13, received a severe cut in the forehead, which healed in a week and had never confined him to the house. Four years afterwards he was frequently wont to carry heavy loads upon his head, and in about a month subsequently he was suddenly seized with spasms of his hands and legs, which lasted for a couple of minutes, but were unaccompanied by any loss of consciousness. These spasms at first occurred once a week, but they gradually increased both in number and severity until they appeared once or twice daily, and continued several minutes. The hands and feet were enspasmed, then followed an uncontrollable jactitation, then the complete fit; and it was only in the middle of the paroxysm that the patient became wholly unconscious of external objects. The attack was sometimes preceded, sometimes followed by headache. It never occurred during sleep, nor in bed, and he always ascribed it to exertion. The nape was blistered, the bowels were kept gently open, and calomel was given until his gums grew tender, but the disease pursued its course without alleviation. The sulphate of zinc was then given, and gradually increased until four grains were exhibited every three hours; under the action of which he recovered. The 259th history may be considered a severe case, and in the 257th epilepsy is manifested in its most aggravated form.

A washerwoman, aged 40, who had borne a child twelve years before her admission into hospital, since which she had never been regular, was attacked with epilepsy, after suffering severely from headache for a considerable period previously. The right temple had been always referred to as the seat of pain during these attacks of headache. She was bled and cupped, but no obvious relief followed, a second paroxysm having occurred, which reduced her to a state of complete blindness with dilated pupils. At first sensation remained unimpaired, her dejections were passed with consciousness, and she answered questions well; yet she was sometimes found to wander a little, she slept much but without stertor, and ultimately she articulated badly, her conversation became incoherent, her sensibility obtunded, her strength oppressed, and she died in less than a month after the supervention of the first paroxysm.

A carpenter, aged 33, was seized with his first fit of epilepsy, while engaged in talk with another man. He had frequently complained of nausea, giddiness and a peculiar sense of sinking at the pit of the stomach: but he never acknowledged headache, nor had he ever vomited. He was brought to the hospital in a state of insensibility, but without any detectable symptoms of paralysis. He was bled, leeched and purged. On the following morning he was convulsed for the first time, and after that the convulsions returned frequently in a very severe form. When seen by the author "he lay tranquil; respiration 25; pulse 64; skin moderate." At times he gave a kind of jerk in his elbow or arm, particularly on the right side: he had frequently a violent movement of all his limbs, such as required four persons to hold him. Pupils contracted and immoveable to light; he never opened his eyes willingly; his appearance was pallid and sunk. His head being shaved had cold applied to it, and a cathartic enema was given. Next day—

"He remained much in the same way, lying in general still and tranquil, and when roused seeming sometimes to know his wife, but was unable to speak, and scarcely able to swallow, occasionally crying out violently till four o'clock in the morning, when he became very violent, crying out, and throwing himself about in bed, requiring several persons to hold him still: he afterwards became tranquil, but apparently more drowsy; he once or twice retched a little, but vomited nothing; his bowels open freely; all the evacuations were passed in bed.

I saw him at eleven o'clock. He was lying as in a deep tranquil sleep, on his back, looking pallid. Respiration 24: pulse 80, soft; skin soft; hands and feet rather too cool. I stood by his side some minutes, and he never moved. When I felt his pulse it seemed to rouse him, as he soon began to move about in a restless manner; then cried out very loud, threw himself about, moving all his limbs, and lying on his side with his legs drawn up.—When his wife called him several times by his name, he said in a loud voice, 'Well!'—but this was the only word we could obtain. His pupils were more dilated than yesterday, and sluggish. On the whole, the powers of life seemed reduced, and he was more comatose.

Admoveatur Emplast. Cantharidis inter scapulas, et Cataplasm. Sinapis pedibus.

24th. After the visit yesterday he seemed on the whole more composed and inclined to sleep. About four o'clock this morning, and again at five o'clock, he had most severe fits, described as quite of an epileptic character; his face was much convulsed, of a purple colour; and foam and blood passed from the mouth; at ten o'clock to-day he had another similar attack.—He appears better; lies frequently with his eyes open; and has several times so expressed himself, as to leave no doubt that he understood questions: he has this morning twice intimated, without speaking, his wish, to pass urine, and that passed is loaded with pink sediment. Pulse 80, rather weak, and not so strong as the beating of the heart at the scrobiculus cordis might lead us to expect. Respiration 20: pupils contract and dilate, though slowly, by the light of the candle: he moves every part as yesterday, but more naturally, and yawns frequently like one waking from sleep.

25th. His head had been kept quite cool: the blister has risen very well on his shoulders. He has been tranquil almost all night, and has latterly become very sensible, so that he answers questions distinctly, though slowly: he says he has scarcely any pain in his head; but his tongue is much swollen, and seems bitten at the edges: bowels not opened. Pulse 80, weaker: respiration 20. He has eaten a good deal of arrow-root. His manner is still quite dull, though he opens his eyes, and has been awake for several hours.

He did not sleep from five o'clock on Monday morning May 25th, till Wednesday the 27th at four o'clock; but became towards the evening of the 25th, after I had seen him, raving and delirious, so as to run about the whole neighbourhood half-naked.

On Tuesday the 26th he was taken to the workhouse, where he remained quite deranged, occasionally requiring restraint, till the Sunday week following, when he became sensible almost suddenly, and made anxious inquiries where he was, having known nothing of his removal from home, and retaining no recollection of anything which had happened

August 2nd. He has been for some weeks able to return to his work, but has almost constantly experienced head-ache, particularly over the forehead, and several times in the day is affected with a slight dizziness and cloud before his eyes, with loss of memory and absence of mind, so that he is obliged for some moments to cease from his work: he sometimes talks in an unconnected manner; his countenance is pale; he has not the slightest paralysis of any part, and has had no return of a decided fit: he complains much of pain at the pit of the stomach and nausea.

I ordered him to rub the ointment of the tartrate of antimony on his neck, and regulated his bowels; but I shortly afterwards lost sight of him." 525.

But, however this disease may be thus diversified both in its premonitory and attendant symptoms, the period of its duration is in all cases short, being seldom above thirty, and generally only a few minutes in duration, After the fit has passed the patient usually falls into a deep sleep, approaching to coma, from which he awakes unconscious of every thing which occurred; but he is often afflicted for days with severe headache, sometimes temporary palsy or delirium follows, and in some cases a death-like sopor succeeds, in which the patient lies immersed as in a fit of apoplexy, for days or even weeks. The mind invariably suffers more or less after repeated attacks of this malady, and it is by no means uncommon to be able to trace many cases of idiotism and insanity to violent and repeated paroxysms.

We as yet know but extremely little of the proximate or even the remote cause of epilepsy. That its seat is especially in the nervous system there can be no dispute, but so is that of hysteria, neuralgia and chorea also; yet these several diseases are very distinct and distinguishable from epilepsy. How far this dissimilarity arises from the nature of the exciting cause, or from the peculiarities of the excited organ, has not been ascertained. In almost all the cases recorded in these *Reports*, and they amount to 22 in number, cerebral congestion was tolerably evident upon dissection; but that congestion evidently arose from very different causes. In no fewer than eleven cases either the skull-cap or membranes of the brain, or brain itself were in a morbid state. In seven of these the cranium was more or less diseased. Thus in the 258th case there was an excessive bony deposit over the sagittal suture; in the 259th the calvarium generally was "at least three times the natural thickness, and the frontal bones about their centres encroached much on the cavity for the brain;" in the 260th the skull was very thick and irregular, in addition to a diseased state of the arachnoid, and portions of the cineritious substance; in the 261st the skull was at least half as thick again as usual and very heavy, and the kidneys were pale and scabrous; in the 263d there was, as in the 258th case, an excessive bony ridge along the course of the sagittal and also of the coronal sutures; in the 265th case the cranium was thick, externally irregular as though embossed, and internally presenting two convex prominences, two inches long and one inch wide, which produced corresponding depressions on the anterior and middle lobe of each hemisphere of the brain; and in the 266th case the skull-cap had been very seriously injured by a fall; since which the patient had been subject to epilepsy. In the 265th and 269th cases there was a fungoid tumour attached to the dura mater; and in the 270th the longitudinal sinus was obstructed by an exuberant growth of glandular structure. The 272d case depended on a deficient action of the uterus; and the 273d arose from abdominal irritation.

"The tangible source of irritation is often within the brain itself, or its membranes, or its bony parietes: at other times we are unable to trace any other appearances than such as would mark a degree of congestion in that organ. Sometimes we infer from the symptoms, or deduce from appearances after death, that the attack has depended upon some distant source of irritation, as the uterus, or the intestines; and there is still so great a similarity between those attacks produced by distant irritation, which leave scarcely a trace upon the brain itself, and those which depend immediately on cerebral disease, that we must suppose that the state to which the organ is brought, in order to produce the attack, is nearly the same in both. It is probable that there is an original formation of the brain, which renders one individual more liable than another to the irritation producing epilepsy; that in such brains, comparatively slight irritations, and such as would produce little disturbance under ordinary circumstances and a more healthy original organization, give rise to the epileptic attack; but that there are sources of irritation so overwhelming that scarcely any brain can withstand them; such, for instance, as important changes in the skull or in the brain itself and its membranes; and when once that irritation has been excited by any cause whatever, the brain becomes more liable to its renewal; and I believe that almost always, during the epileptic paroxysm, either as a cause or an effect, sanguineous congestion takes place within the brain.

As far as I have been able to infer from my own observation, I should say that the organic causes of epilepsy, connected immediately with the brain, are more frequently such as affect its surface, than such as are deep seated in its substance. Thus we find that morbid growth, taking place in the skull, showing itself by a thickened heavy state of the bone, or by a roughened surface either internally or externally, or a remarkable prominence in the natural projections at the base, is often associated with epilepsy. Slow changes, producing a thickened condition of the membranes, will not unfrequently be found attendant upon epileptic attacks. Tumours pressing on the surface, or amalgamated with the cineritious substance, will also be found in cases of epilepsy: and these observations connect themselves in some way with the hints thrown out at page 46 and 381 respecting the apparent dependence of spasmodic action, in many cases, upon injury done to the cineritious substance. It is an idea entertained by Dr. Foville, that the cineritious is the more active part of the brain generally, with regard to all its functions; and that the medullary part is more particularly employed in the conveyance of the motions and sensations, or whatever else may be acted upon or produced in the cineritious part. And supposing for a moment this to be the case, we might expect that lesion of the cineritious substance would produce disordered action in that part; and that such action might be transferred to the distant parts of the body, producing disordered and involuntary motions: whereas, if the great injury were done in the substance of the brain, the means of communication with the active part being cut off, paralysis might result, more or less mingled with convulsion, in proportion as the cineritious substance is more or less involved." 515.

Epilepsy is occasionally accompanied with apoplectic, sometimes with paralytic symptoms.

(253.) J. C., aged 59, was much depressed in mind by misfortune, and became affected with headache, giddiness, and loss of appetite, which gradually increased, until on entering his house one day after severe fatigue he fell down senseless, out of which he recovered by having a little spirit administered to him. Next day he was bled and purged, and felt much better in consequence; but in less than a week afterwards his disorder returned, for which he was again bled, purged and had cold applied to the head with the same effect. Two days after, however, a third fit occurred of a more violent description, which rendered him almost completely unconscious, and which was followed by several others. He was again bled and had a blister applied to the nape, but he did not rally as before, and he died without having any fixed paralysis. The dura mater was slightly vascular, but the cerebral substance was " more remarkably perforated by vessels than I almost ever before saw"—especially " in the upper part of the hemispheres, and most of all on the left side. The cerebellum was in the same state and more particularly on the left side."

From the obvious apoplectic character of this case of epilepsy the practitioner was induced to employ his lancet, and certainly twice with a good effect; but the Doctor believes both from his experience in similar instances and from the epileptic features of this case having been the most predominant, that local depletion, in conjunction with the other remedies employed, and tonics might have been productive of better consequences; and this opinion is countenanced by the dissection of the brain, in which there were more vestiges of prior than present congestion; for, speaking of the divided vessels above referred to, it is said that they "did not appear as if they contained any great quantity of fluid blood—the longitudinal sinus scarcely contained any blood; the smaller sinuses a small quantity in a fluid state—the choroid plexuses were remarkably pale; and the large veins, which run along them, contained no blood, looking like white worms or threads."

(271.) A stout well-made young soldier, after being on guard, was suddenly seized with a fit of epilepsy, in which he was not convulsed, but foamed at the mouth and became black in the face. This fit was followed by many of the same character, for which he was occasionally bled and purged; but he never acknowledged the existence of pain in his head, or indeed in any other part of his body. Eighteen months after he first took ill, his right leg and loins became numb and motionless, and in 24 hours after his left leg was similarly affected. His urine and stools were voided unconsciously, and these paraplegic symptoms continued for eight months in spite of cupping, setons, issues and other remedies. Power gradually returned to the left leg, and then sensation; but the faculty of motion alone was restored to his right extremity. In this state he was dismissed from Chatham Hospital, and after having been frequently cupped in St. Thomas's, he ultimately was admitted into Guy's. His fits at this time were less frequent than formerly, and he had a brief notice before the rectum or bladder discharged their contents. His left leg moved very imperfectly, but preserved its sensibility unimpaired; while the right enjoyed full power of motion, but might be pricked without feeling, and "if the needle were pushed in deeply a slight convulsive action only of some muscles occurred, not at all like a retraction of the limb from pain."

In this case the Doctor suspected that some organic change was taking place within the cranium, but the deterioration of sensibility in one part and of motive power in another renders it difficult to fix its precise seat, or to predicate its exact nature. Both legs were similarly palsied in consequence of severe epileptic fits excited by tapeworm, in the 273d case; but the use of the left returned after a few days; and in both the 261st and 262d cases partial palsy was intermingled with the epileptic symptoms.

As in all other complaints there can be no doubt but that also in epilepsy the cure will materially depend, both for its means and probabilities, upon the nature of the exciting cause. If it arise from mere functional derangement, whether of the brain itself or of some distant organ with which it sympathises, medicine can promise more than when structural disease of either the cerebral tissue, investing membranes, or osseous covering is the exciting agent. As has been already observed, the vessels of the brain are generally in a congested state, and as this congestion, whether it be sympathetic or immediate, is a morbid and morbific condition, it is indispensable to ply it with the appropriate remedies carefully employed. For this purpose cupping on the nape, setons, issues and perpetual blisters are usually had recourse to; and, although our author disapproves of free depletion as a general measure, he speaks in terms of approbation of the local removal of blood in moderate quantities, when evident signs of congestion are discoverable. Dr. Prichard recommends large issues along the vertex. The Doctor has not had any experience of their effects; but the tartar emetic ointment he advises to be rubbed upon the scalp or neck, until an abundant eruption is produced. A slight mercurial action has occasionally been followed by relief, as have also brisk mercurial purgatives; all the animal functions should be carefully attended to, as their derangement has been found strongly to aggravate and not unseldom wholly to induce the epileptic paroxysm. With this view the mineral tonics are the most promising, and of these the sulphate of zinc, the different forms of iron, the arsenical solution, and the nitrate of silver are the most powerful. The shower bath is often a strong auxiliary, but where symptoms of much cerebral congestion are present, it must be employed with caution. In hysterical epilepsy the entire class of diffusible stimuli have been tried with advantage.

The Doctor has been frequently led to observe that where the disease is mainly or wholly dependent on cerebral mischief, the first symptoms of attack manifest themselves in some distant parts of the body; hence the great importance of attending in all cases of epilepsy, but more especially in such, to deranged although they may be distant functions. In the two following histories it will be seen that the alimentary canal was in one, and the uterus in the other seriously disordered, and how attention to these organs overcame the nervous symptoms which they had induced.

(272.) A young female, who had never menstruated, but who often complained of pains in her loins and lower region of her abdomen, became subject to fainting fits, in one of which she struck her head rather severely against a stool. A fortnight after this accident unequivocal epileptic symptoms appeared, complicated however with globus hystericus, and occasional sobbing. A good drastic purge brought away several unhealthy stools, after which headache supervened; for which ten ounces of blood were cupped from the nape, a stimulating foot-bath was ordered, and aloetic wine with camphor mixture were taken every eight hours. On the 26th she felt better, and her fits had less of an epileptic character; on the 27th she had one fit only, but not having had any stool some castor oil was given, and as she complained of severe headache, giddiness and sickness of stomach, a draught of the tinct. helleb. nig., aq. puleg., and syrup. aurant. was prescribed twice daily. By the 1st of December the fits had disappeared, but she occasionally felt faint, and shortly afterwards by persevering in the same medicines she got quite well.

(273.) A young man, who had been for several years subject to tænia, against which he was in the habit of taking turpentine, having experienced severe intestinal pain, had recourse to his usual remedy; but for five days no effects followed, strangury occurred, and he experienced several fits of epilepsy, under which he lost the power of both his lower extremities. His fits were frequent, and on the day of his admission he had suffered six. The turpentine was persevered in with the effect of bringing away large portions of tape worm; for about a week five grains of blue pill were taken every night, but they were suspended on his mouth becoming sore; balsam of Peru was afterwards ordered, and when he left the wards he remained free from fits, and was slowly regaining the use of his extremities.

*Convulsions.* What are usually called convulsions in children the Doctor regards as of an epileptic character, while he admits that they may appear in the very severest form, without ultimately establishing the habitual and regular disease. These convulsions arise from as many and as varied causes as epilepsy—such as fluids effused upon the brain, vascular turgescence, tumours attached to the membranes, or imbedded within the substance of the brain, the excitement occasioned by such febrile diseases as small pox or scarlatina, internal irritants, as dentition or worms, and external ones as injuries or burns.

"These exciting causes of the convulsions of children will be perceived to resemble very much the usual exciting causes of epilepsy,—allowance being made for the irritability of the infant's frame; nor are there any circumstances in the progress of the attack which enable us to draw a distinct line between the two conditions, except the very frequent occurrence of convulsions in children, without leaving either a tendency to return in after life, or any trace of the attack when it has passed away. I have seen children for six or eight hours convulsed and senseless from abdominal irritation recover completely in a few hours, and grow up to manhood without a symptom of disease. I have known a child from the time it first began to cut its teeth, suffering unceasing convulsions, despaired of from day to day, and from week to week; yet, after the lapse of several months, recover completely on the appearance of its molar teeth. It does, however, on the other hand, occasionally happen that repeated convulsions impair the faculties, or are followed by a tendency to epileptic seizures during the remainder of life, or leave traces of paralytic infirmity of a more or less durable character. In such cases, it is probable that slight lesions have been produced in the brain during the excessive congestion of the convulsive paroxysm, or that the vessels have been so distended as to be unable to recover their healthy tone and condition." 554.

*Tetanus.* Whether we consider the apparent slightness of its exciting cause, in contrast with the frightful severity of its attendant symptoms; or the almost certain death to which it leads its victim, with the energetic efforts which have been made to vanquish it, there are few diseases more truly formidable than tetanus. Were tetanus an exclusive consequence of a wounded nerve, or were every wounded nerve to be succeeded by tetanic symptoms, the etiology of this disease would be less clouded; but when the largest and most important nerves may be irritated, incised and severed without inducing tetanus; when tetanus may appear not when the nerve is wounded, but years after it has healed; and when tetanus may occur without any known cause, and after baffling every remedial effort destroy life without leaving one post-obit trace of either its cause, seat, or character—we are doomed to struggle with an enemy of so subtle a nature, as to make its attack from a quarter unperceived, with a ferocity too often superior to control, without giving the faintest premonition, or furnishing the vanquished with any autopsic evidence of whence it came, or how it killed.

The Doctor describes the approach, progress and result of this disease in the following accurate and graphic style:—

"At first the patient suffers a slight feeling of indisposition; he thinks he has taken cold, and perhaps he has; the muscles of his neck grow stiff; he feels an obstruction in swallowing; he fancies that his throat is sore; he finds it difficult to open his jaw, which is often first perceived when the physician wishes to see his tongue; the muscles of his face contract; his brow becomes wrinkled; his eyes are drawn by the action of the orbiculares; and the corners of his mouth are pulled down, particularly in the effort of opening his mouth. He now feels other muscular spasms; he is drawn for a moment backwards; the abdomen becomes rigid; the pulse is often hurried and irregular; the respiration is embarrassed; he complains of pain at the pit of his stomach, increased to agony on each recurrence of the spasms; he is bathed in perspiration; he is marked with torment; the spasms act upon him with increased violence; he is worn out with suffering, he becomes delirious, and he dies: or a more sndden termination is granted to his miseries; the disease fixes on the muscles of respiration, and brings on instantaneous death.—All this may be the work of four and twenty hours;—it may be the work of as many days. Sometimes, on the contrary, the disease remains mild for several days, abates, and passes off." 555.

When the attack has been occasioned by external injury, the nerves of the violated texture are generally found more or less vascular and reddened; but when it has appeared without any known cause, it not unfrequently happens that neither the brain, nor spinal cord betrays the least vestige of derangement. This is well seen in the history of the following case.

(276.) A boy, aged 15, got both his heels wounded by a circular saw, and his left humerus fractured, after which he became very irritable, took what he called a sore throat, and symptoms of trismus came on. His tongue was protruded with great difficulty, his neck was stiff and retracted, he complained much of a pain at the pit of his stomach, the muscles of his abdomen were hard, and his pulse was 127, wiry. A scruple of musk, a dram of æther, and five grains of the subcarbonate of ammonia were given in peppermint water every three hours, a sinapism was applied to the feet, and a stimulant enema was injected. The wounds were dressed with spirits of turpentine, and brandy and wine were given with his nourishment. Towards night he became more irritable, and a dram of laudanum was administered as an injection every quarter of an hour, until two ounces were consumed; but no alleviation was procured, and he died in forty hours after the first appearance of his disease. The dissection was conducted by Mr. B. Cooper and Mr. Key, but "no diseased appearances were discoverable." The brain, the cord, the ganglia, the sympathetic, the plexuses and all the nerves, which could be traced, were all equally and perfectly healthy. The lungs were somewhat congested, the large veins near the heart and the right auricle of this organ were distended by coagulated blood, the gall bladder was filled with bile, there were some small calculi near its orifice, and the small intestines were much contracted.

With curative indications so feebly developed, it is not marvellous if our treatment of tetanus should be empirical and its success uncertain. In the periodical publications of a single month within the last two years, says the Doctor, cases were brought forward by four different practitioners, which had been successfully treated by four different remedies—the subcarbonate of iron, mercury, caustic potash to the spine, and general bleeding; and scarcely does a year pass, which does not boast of some new curative plan, or some new successful specific. The 275th case must be read with interest in reference to this point.

A youth, aged 15, was attacked with difficulty in opening his mouth and swallowing, whether from exposure to cold during his employment as a brick-maker, or from a cut received by a piece of glass on the thumb about six weeks previously, which had quite healed, was uncertain. A draught, composed of æther, decoction, and tincture of bark, and laudanum, was ordered every two hours, and a purging enema was administered. On the next day (31st July) there was no change. He perspired profusely, his temperature was 105°, his pulse 130, and he complained of painful spasm at the pit of the stomach, for which a blister was applied. 1st August. No power of voluntary motion except in arms; neck and back retracted, legs extended, and all his muscles were successively in spasmodic action. When raised from the bed to change the sheets he stood "stiff and erect, touching the ground only with his toes; his neck stretched to its utmost, and forming part of the curve into which his body was thrown backward; he was much oppressed; his breathing and articulation were difficult." 2d. No improvement, in consequence of which cold affusion was tried. Being placed in a tub, a large bucket of water, at the temperature of 60°, was thrown over his head, and repeated twice after considerable intervals. 3d. Each application of the water gave temporary relief, and both the pulse and temperature were slightly reduced by it; but he passed a noisy restless night, and abused the attendants because they would not repeat the bath. 4th. Four baths had been since given with transient benefit, but as the spasms were considered even more violent than formerly, and as the benefit derived from the affusion was very fleeting, it was again resolved upon to change the treatment and substitute tobacco injections. Half a drachm of the leaves of tobacco was accordingly infused for ten minutes in half a pint of boiling water, and formed into an injection. In ten minutes the pulse became 140 and irregular; in 20 slight nausea was produced and the pulse rose to 160; in 30 the nausea continued, but the pulse fell to 96; and in an hour the pulse stood at 100, and the nausea was going off. 5th. The glyster had been twice repeated, and during this day was again administered three times; but not finding that it had arrested the progress of the disease in the least, it was resolved not to harass the patient longer with the injections. One ounce of strong mercurial ointment, one drachm of opium, one of camphor, and one of oil were now made up into an ointment, of which one drachm was rubbed in every second hour; and five grains of calomel were given in the form of pill, and one drachm of laudanum every four hours. (6th.) Passed a better night, and thought himself more easy. (7th.) Passed a worse night, his back was bent backward, every muscle was stiff but not so painful, and he looked much worn by the disease. (8th.) Had a tolerable night, but although his mouth and throat were becoming sore, the countenance was dejected, the breathing short, the pulse rapid, and no one symptom was improved. Mercurial friction was, therefore, laid aside, and a scruple of musk with five grains of ammonia were given every two hours. (9th.) Bowels irritated, much pain of head, neck and abdominal muscles, back spasmodically bent every four or five minutes, mind very irritable, he drank much, but refused to eat even jelly. As half an ounce of musk and one drachm of ammonia had been given without producing any good effect, these medicines were abandoned; and as he was evidently sinking, no further remedy was proposed, but as much nourishment was given as would be received. (10th.) Passed a restless night. Had taken an ounce and a half of port wine every hour, and had eaten some jelly and oysters. The pain in the back and spasms, however, continued, deglutition became still more difficult, during the day his intellect wandered for the first time, as evening approached he grew weaker and dyspnœa became excessive, and during the night he was delirious. (11th.) This morning the delirium continued, as the night advanced it increased, and shortly after six on the morning of the 12th he expired.

There were, thus, four distinct remedial systems made to bear upon this patient, and although the shortness of the period during which each was tried may render these experiments objectionable, the inefficacy of each was ascertained with tolerable certainty. The calomel and opium at first seemed to do good, but by the time that ninety grains of calomel, three ounces of mercurial ointment, and two ounces of laudanum had been employed, all the symptoms were worse, and the patient was evidently sinking. The cold dash gave relief, and quieted the patient's irritability both of mind and body more effectually than any other remedies; but still the spasms continued unabated, and the temporary respite, which followed the affusion, was invariably succeeded by symptoms of reaction as formidable as ever. The tobacco injections were obviously injurious, by exhausting the powers of life; neither the musk nor the ammonia, the æther or the bark gave the promise of any certin good; and, after making so many fruitless efforts to procure aid from medicine, it was at the last found impracticabe to achieve more than sooth (with wine and cordials) the last moments of an existence which it was impossible to prolong.

General bleeding is reprobated as only calculated to increase irritability and diminish strength; leeches and other locally depleting agents are recommended, as having been applied with advantage; but our author has not been able to discover that vascular condition of the medulla oblongata, or spinal cord which has been witnessed by some. The blood, which the Doctor would remove in this affection, he would not abstract with the view of reducing the general powers or of weakening the general circulation; but to relieve the patient of that congestion, which is supposed by many and has been shown by some to exist about the column of the spinal cord. This local depletion he would accompany with other measures. Notwithstanding the very conflicting evidence of Currie and Morrison, he thinks favourably of cold affusion. It is reported to have cured cases both of the idiopathic and traumatic character, and in the history last given its operation, although merely palliative, was more soothing and conciliating than that of any of the other remedies employed. Strong opiates and tobacco injections he considers useless, if not injurious; but he speaks in terms of decided preference of the tonic stimulants. "I scarcely remember," he observes, "to have seen one patient recover to whom tonics, chiefly bark and generally stimulants as wine and ammonia, had not been pretty liberally administered." In the 274th case the efficacy of this tonic plan was fairly tested and answered favourably. One hundred and eighty leeches, no doubt, had been applied to the spine, and some may ascribe to them considerable influence; but the relief, which they gave, was only transient, and the Doctor fancies that little ground was gained until the tonic treatment had been fully established. It will be seen that the sulphate of zinc was at first given in two grains, but was afterwards increased until eight grains were ordered every six hours.

(274) A boy, aged 14, was bitten in the leg by a dog, a week after which he felt unwell; after a few days more he experienced some stiffness in the jaw, pain in the chest and abdomen, and the wound, which had hitherto been doing well, became foul, and in the course of three weeks he was admitted into Guy's Hospital with decided tetanus. A pill, composed of one grain of calomel, one grain of opium and a quarter of a grain of tartar emetic, was ordered every four hours together with an aperient. On the 14th the pills were repeated four times daily, and a blister was applied to the wound. 15th. Symptoms of opisthotonos appearing 40 leeches were applied to the spine, the pills were continued, and the blister was dressed with cantharides ointment. 16th. Trismus more advanced, and abdomen more rigid. Twenty leeches to spine, pill continued, and a black draught given. 17th. 20 leeches again applied; other medicines repeated. 18th. Appears better—20 leeches again ordered, pill continued twice daily, and two grains of the sulphate of zinc given every six hours. 19th. The black wash was applied to the wound, 20 leeches were applied to the spine, the zinc was continued in three grain doses, the pill was repeated at bedtime only, and two grains of the extract hyosc. were given every six hours. (24th.) Twenty leeches had been since applied, the zinc was increased to eight grain doses, and the pill was continued at night, but without the calomel. The patient sleeps well—abdomen nearly natural, mouth can be opened more easily, no pain any where. (Dec. 1st.) Sat up. (5th.) Convalescent. (7th.) The zinc was continued every eight hours. (8th.) Wound in the leg healed. (11th.) Left the hospital quite well.

Dr. Elliotson has been more than once successful with the carbonate of iron, and Dr. Bright's experience of the sulphate of zinc in chorea, neuralgia, hysteria and epilepsy leads him to conjecture that no general tonic may be found serviceable in tetanus, which these affections resemble in more respects than one.

"Hysteria, chorea, and epilepsy are all relieved, and in some cases cured, by tonics; and the two former are frequently benefited by stimulants. Epilepsy and hysteria, and even chorea, occasionally approach for a short time very closely to tetanus in their symptoms;—and why should we not seek the remedies for one, out of those most useful in another? Similar analogies would lead us to pay the strictest attention to the condition of the bowels, and by procuring daily full evacuations, to satisfy ourselves that no accumulation either of the remains of alimentary matter or of vitiated secretions takes place; but I cannot help considering the state of irritation which has often been kept up in the alimentary canal by drastic purging, as likely rather to prolong than relieve a disease in which the irritability of the system is so great.—When any local injury can be discovered capable of keeping up irritation, it is obviously a great point to remove it as quickly as possible, or the irritating action of the part should be changed; and with this view, the practice recommended by applying blisters to the wound, as well as that of dressing it with mercurial ointment till a more healthy action is induced, appear amongst the most promising remedies." 557.

"I know of no remedies," he remarks, "which seem to go more completely hand in hand, in the cure of nervous irritation, than sulphate of zinc and carbonate of iron. Borne out, then, not only by the analogy of chorea, in which experience has been abundant and convincing, but by the apparent result of much more limited experience in the disease itself, we seem to have a clue at least to guide us to a treatment as rational as any which has hitherto been adopted." 563.

In the 258th case the surface of the anterior lobes of the brain was diseased, but the author is inclined to ascribe this appearance to some prior mischief; and in the 259th history an encysted abscess was discovered in the cerebral substance, which lay underneath that portion of the scull, which had received the blow. But as the appearance of the tetanic symptoms preceded the formation of this abscess, supposing the abscess to have been an effect of the blow, it may probably have increased the nervous disturbance, but could scarcely have exerted any more primitive or essential influence.

*Hydrophobia.* Many of the observations, with which our notice of tetanus has been introduced, are equally if not still more forcibly applicable to hydrophobia. Indeed, in many very interesting respects these two diseases, which are so usually treated of separately, and considered perfectly distinct, remarkably correspond. They are both strictly nervous diseases not only having their seat in some portion of the nervous system, and exhibiting the same nervousness of symptom, but any medicines, which have hitherto been found in the least to influence them, are such as acknowledgly possess some peculiar power over the sentient system. They are both dependent on similar causes—nervous irritation—whether that irritation arise from the immediate injury of some nervous tissue, or the mediate agency of some distant excitement. They are both idiopathic, as well as traumatic; for, in our opinion, it has been tolerably well demonstrated that cases of real hydrophobia have occurred in persons, who had never been bitten at any period of their lives by a rabid animal. They are both attended by many symptoms equally common to either,—such as violent, irregular and repeated spasm, difficult or impracticable swallowing, pains in the head, abdomen and back. They are both equally uncertain in their occurrence, after the cause which usually excites them has been applied; for hydrophobia no more certainly succeeds the bite of a rabid animal in all instances, than does tetanus a lacerated wound. They are both alike rapid in their progress, active in their character, intractable in their obstinacy, and fatal in their career; and lastly, they are both almost, if not altogether purely functional diseases; so that after death the knife is unable to detect any organic mischief at all adequate to the symptoms of disturbance which were manifested during life. It is, no doubt, true that an invincible dread of water is a very ordinary feature of hydrophobia, which is not usually met in tetanus, and that locked jaw and empros and opisthotonotic convulsions occur in tetanus without appearing in hydrophobia. But it is equally certain that these very symptoms, which have been considered so pathognomonic and peculiarizing, are neither inseparably present in their respective affections, nor invariably absent from their opposites. These opis and emprosthotonotic movements, which appear in tetanus, have been seen accompanying hydrophobia; and the *terror aquæ*, which marks the latter, has been found in attendance upon the former. These facts—and others might be advanced, such as the fact of hydrophobia succeeding to a bite inflicted by a dog under excitement, but not mad—render it probable in our eyes, at least, that there may be less etiological connexion between the rabid state of the animal which bites and the disease of the person bitten, than between the structural violence committed by the bite itself and the subsequent tetanic symptoms. We say *probable*, because we are not yet prepared to maintain the precise identity of hydrophobia and tetanus, nor would we select the present as the most appropriate place for doing so, were we prepared; but we certainly can discover very striking resemblances between these two diseases, and we are not aware of any very serious practical evil, which could issue from the adoption of such a theory. It may, indeed, be argued that if the poisonous saliva of the rabid animal have no effect in inducing hydrophobia, how happens it that hydrophobia so frequently succeeds to the bite of an animal so circumstanced? or it may be objected that the theory, which supposes such a thing, would render all local measures, intended to prevent absorption of the poison, useless, and would thus, were it badly founded, tend to endanger the patient's life. These and other objections may be urged which it were now irrelevant to reply to. Our only object has been to call the attention of the reader to a similarity, which the facts we have adduced are sufficient to establish; in order that future observers may make further inquiries upon a question, which appears to us deserving of investigation.

The Doctor has favored us with six very interesting cases of this frightfully fatal malady. By these it would appear that several of those persons who were attacked, had been very irregular in their habits, and were highly predisposed to suffer from any such nervous distemper—that the wound had in general been inflicted upon some uncovered and, therefore, unprotected part of the body—that the first sensations of pain were generally referred to the wounded part, and that the latent period, which intervened between the infliction of the injury and the supervention of the specific symptoms, varied from six weeks to two months. Some have fancied that cases have occurred, in which the disease did not break out for years after the wound had been received, and the author believes that a shorter period than six weeks seldom intervenes between these events in any instance. In what state the poison exists during this period of latency—whether it be slowly but impereeptibly saturating the constitution, and thus preparing it for the sudden and complete development of the evil; or whether it be totally inactive, either in consequence of its own weakness, or the insusceptibility of the system; or, lastly, whether it require a certain period to be absorbed, and must be in the circulation for a certain time before its specific influence can be exerted —is not only unknown but seems likely to remain so. It appears probable to the Doctor—

"That for some considerable portion of that time it is confined to the neighbourhood of the primary wound; and the secondary local action, which so often precedes the appearance of symptoms, leads to the belief that some excitement of the part is necessary, or at least frequent, before the constitutional disease is developed, or perhaps produced.—These considerations lead to one very important practical inference,—that the excision of the part will probably prevent the disease, though performed a considerable time after the accident; and to this we can of course place no limit, except the actual appearance of the disease: when that has once established itself, we have in a case which is above recorded, a strong evidence of the inefficacy of an operation." 605.

The case here alluded to is the following:—

(282.) A young man had been severely bitten on the back, palms and fingers of the left hand by a strange dog, from which he had been endeavouring to save a child. The wound healed, the child remained unaffected, and the dog was killed; so that no apprehensions of danger were entertained. In less than two months thereafter, however, stiffness about the back part of the head came on, with tightness of the epigastre, shivering and some difficulty of swallowing. These symptoms so rapidly increased in intensity as shortly to dispel all uncertainty as to their real nature, and as every remedy yet tried in this disease had proved fruitless, amputation of the bitten limb was proposed, and the left arm was removed above the elbow. As soon as he had recovered from the shock of the operation, he said he felt better; but considerable hæmorrhage soon took place from the stump, violent convulsions succeeded, priapism came on, the pulse grew weaker, and he died 48 hours after he took ill. There was no disease worth notice in either the membranes, brain, or spinal cord. The membranous lining of the trachea, particularly about the bifurcation, was vascular, and the lungs were somewhat emphysematous, as was the cellular tissue in the anterior mediastinum. The œsophagus was slightly marbled, and near the cardiac orifice of the stomach the mucous lining denoted partial turgescence.

Indeed, the Doctor's observation concerning the inefficacy of an operation after the full development of hydrophobic symptoms is equally applicable to every other remedial measure. Every curative plan hitherto attempted has equally failed, and the only chance of safety, which art affords, consists in endeavouring to prevent the approach and establishment of a disease, which when once developed is no longer within the influence of human skill. It is, therefore, only adding mutilation to misery to amputate the bitten limb after the pathognomonic symptoms of hydrophobia have been clearly and fully manifested, for whether the essence of this disease consist in a poison to be absorbed, or in a morbid nervous action to be enkindled, the period of hope has then expired, and all kinds of treatment, which aim at any thing more than transient palliation, have, at least as yet, been tried in vain. Bleeding was attempted in the 280th case.

(280.) A man, of 52 years of age, had his upper lip bitten by a dog, but he had caustic applied to the wound and it healed. Five weeks afterwards he refused to take his tea and complained of headache, loss of appetite and shivering. Sixteen ounces of blood were taken from the arm, and a purgative was given him; but he passed a perfectly sleepless night, and next day he was much worse. On attempting to eat an orange, he no sooner had it brought to his lips, than he leaped with force out of the bed, and had he not been restrained by the bystanders would have rushed out of the apartment. He was again bled to 40 ounces, which weakened his pulse, but his restlessness, distress of mind, and convulsions continued. He feared the approach of any thing to his lips, he respired ten or twelve times in a minute, he spoke in a hurried and indistinct manner, and he refused to spit, lest it might agitate him. One pint of blood was again taken from his arm, which had only the effect of rendering the pulse imperceptible at the wrist, for a violent tetanic convulsion came on while the arm was being tied up.

He complained of an ardent burning heat in his head, and about the pubis, and said that, dare he drink it, he would give the world for a pint of beer. He continued sensible to the last, but his headache was so intense that he requested those about him to squeeze it forcibly, and he expired within 48 hours after the appearance of the first symptoms. "On questioning the wife it appeared that she did not at all believe that the disease originated from the bite; but she said that she thought her husband had always feared that this disease would be the result, though he had not openly expressed it, being as she said a close-minded man."

In the 283d case hydrocyanic acid was tried.

A middle-aged sea-man of very intemperate habits, who had been bitten by a small dog, of which he knew nothing, was seized with hydrophobic symptoms about four months afterwards. Two pints of blood were removed from his arm in two bleedings, and then he was placed under the influence of prussic acid in ten drop doses every two hours, giving two grains of opium and five of calomel in the intervals. He complained principally of weakness and slight pain about the second dorsal vertebra; every effort to drink was made with great difficulty and aversion, and as soon as the fluid touched his lips he heaved a deep spasmodic sigh, which prevented the fluid from being swallowed, and was invariably productive of intense weakness. Porter seemed to be the fluid which he preferred, and by means of a long tobacco-pipe he contrived to drink nearly half a pint of it and some barley-water. Next day his symptoms were unchanged. He had slept little, complained much of want of air, but said he felt no pain whatever. A fan was employed to ventilate him, but in a moment he was seized with a spasmodic action of the diaphragm as in drinking, and it was laid aside. He had yesterday attempted to drink out of a cup, but when requested to do so again he replied, "Oh! for God's sake, gentlemen don't ye talk of it; indeed I would if I could, but I cannot; it does make me so bad." At this time his pulse was 140, his temperature 98°, his tongue was brown and furred, he was thirsty but less so than formerly, and his bowels were open. He now began to talk incoherently, and shortly after a scene occurred, which we shall beg leave to give in the language of the original.

"I shall never be any more comfortable in this world: O Christ, grant that I may live to see my wife once more!" Then a violent spasm almost overcoming him, he sat with convulsive haste on the side of the bed, and presently was got in a reclining posture in his bed. He here continued his vociferations still more loudly, declaring he had never injured any man, and looking towards the window with extended arm; "Ah! this is the blessed afternoon when God will take me to himself; the day is beautiful, but it will be more beautiful presently, I hope: and thus he raved on incessantly. His wife came; he knew her well, blessed her, and told her to be good. What passed after this I do not exactly know, but he grew more and more violent; and some man coming who said he should not remain in the Hospital, but should go home; he joined in the cry, and declared that come what might he would go home. He rose from his bed, and partly supported by his friends, he tottered like a drunken man through the ward, declaring that he would go home, and giving very severe blows with his fists to several who opposed him. His friends at length seated him in a chair, in order to carry him out of the Hospital; but a little before they came to the steps, he sprung from the chair and attempted to escape from them; he was raving, and it was found necessary to shut the gates to prevent his running naked into the outer court of the Hospital. He was by this time almost exhausted, and sunk back into the chair, still vociferating, till he fell into a state almost of insensibility, his head fell back, and it was scarcely possible to discover in the livid cheeks and the dark parched lips the features of the strong, powerful, I may say healthy man, who but forty-eight hours before was able to fulfil all his laborious occupations, and who but twenty-four hours ago, when brought to the Hospital, still bore scarcely the aspect of disease. He was carried back to his bed; he never recovered to speak again, but suffered some violent convulsions, in which he threw from his mouth some dark-coloured frothy mucus with saliva, and at a little past 3 o'clock he died;—the whole period from the first appearance of symptoms being less than forty-eight hours." 595.

The blood was found remarkably fluid—a little water lay both above and beneath the arachnoid—the pia mater was rather too vascular—there were two bony concretions in the falx—the cerebral substance was firm and rather vascular—the ventricles were unusually dry—between three and four drachms of serum lay in the spinal sheath, but the cord was healthy—the lungs very much gorged with half coagulated blood—a little bloody mucus lay in the pharynx, the posterior surface of which presented a purple blush—the trachea was of a deep chocolate color throughout its whole ramifications—the lining of the œsophagus was healthy, if we except a reddish purple appearance near its termination in the stomach, which presented internally a few star-like bloody spots towards the cardiac and a greenish purple stain at the pyloric end. In the case, which succeeds the one just now given, sugar of lead was tried with the following result.

(284.) A stout middle-aged man, who had for several years complained of a severe cough and occasional giddiness, came home from his club one night very unwell, without having drunk more than a pint of porter. When seen by Mr. Whitmore his appearance was very peculiar, and as he expressed a strong aversion to the opening and shutting of the door, or the blowing of any breath from his mouth, some suspicion began to be entertained that his complaint was hydrophobia. For some time he would not admit that he had ever been bitten by any animal, but he at last acknowledged that one of his hands had been injured by a pug-dog, to which he had been administering some medicine about three months before. As his fingers contained several scars the situation of the bitten parts could not be clearly ascertained; therefore, no local application was employed. From thirty to forty ounces of blood, however, were drawn from the arm, an injection of assafœtida and belladonna was administered, ten leeches were applied to the epigastre and afterwards a linseed poultice. When first seen by the Doctor his countenance appeared anxious, he sobbed occasionally, he expressed the greatest apprehension on opening and shutting the door, he had no pain, his pulse was 88 irregular, he dreaded the approach of what he called "the fit," he said he thought that he could take medicine if it were made up in pills, but on swallowing a table spoonful of liquid he seemed to do it with extreme effort. A scruple of the subacet. plumbi and eight grains of the ext. belladonna were made up into four pills, of which one was taken every second hour, and a large blister was placed between the shoulders. During the night he became extremely delirious and violent, requiring a waistcoat and two keepers, and by eight o'clock next morning he presented a most pitiable appearance, his countenance being haggard, his articulation indistinct, and his mind totally unhinged. He coughed much and threw up, with a retching effort, some brown grumous fluid not unlike coffee-grounds, which seemed to be blood that had been effused and altered in the stomach. The lead was increased to seven grains every two hours, and as he began to swallow at this period with tolerable ease, forty drops of Goulard's extract were exhibited on sugar; but he died in the night—about 57 hours after the period of the first visit. Five hours afterwards the body was examined, when the scull was found thick, but the brain healthy in every respect. The œsophagus was quite natural—so was the larynx, and the trachea contained nothing unusual, save a quantity of mucus which had so accumulated at the bifurcation, as completely to clog up the bronchi, which were at this part somewhat congested. The lungs were dark-colored and rather doughy to the feel, not crepitating very naturally when compressed. They adhered firmly by old connexions, and there was no fluid in the pleural cavities. The heart and large vessels were healthy, and there was no more than the ordinary quantity of serum in the pericardium. The liver was soft and of a drab-green colour, and the gall-bladder was full of healthy bile. The stomach was large and relaxed, full of a greenish-brown fluid, and shewing several distended bloodvessels. The intestines, kidneys and spleen healthy, if we except a patch of cartilage on the convex surface of the last organ.

In these three most interesting cases, then, have we three most different modes of treatment—depletion, sedatives and tonics—but in none would it appear that medicine exerted any obviously good effect upon either the duration or the severity of the symptoms. In consequence of some traceable analogy between hydrophobia and some other nervous disorders, the Doctor seems inclined to hope, that something may be done through the cordial and tonic remedies, which he has found most beneficial in the latter, aided by cupping, leeches, moxa and blisters to the nape and spine. The actual or imagined dysphagia, however, which marks this affection, renders the exhibition of internal medicines always difficult and often impracticable ; and the restless irritability and malaise of the patient not unfrequently obviates the application of external agents. If injections can be administered they are the least obnoxious forms, in which internal medicines can be given ; but no slight difficulty is occasionally encountered in persuading the patient to submit to more than one or two introductions of the pipe.

In all cases admitting of the operation in proper time, the bitten part should be freely dissected out and afterwards cauterized; and as it has appeared that the application of cupping-glasses to poisoned wounds in all instances retards and in many wholly prevents absorption of the poison, it is advisable to employ them both before and after the excision of the bitten part, when its situation is such as will permit their use. As in tetanus, so is it in hydrophobia—the knife can do nothing after the appearance of the disease ; therefore, unless it can be employed immediately on the receipt of the injury, its subsequent application will be fruitless.

From none of the dissections, which have yet been made, is it manifest that any organic lesion of consequence is established by hydrophobic action in any vital part.

" In the brain (says our author) the utmost morbid appearance is some trace of such chronic derangement as may possibly have predisposed to the excessive irritability attendant on the disease, and some marks of vascular turgescence, even leading to slight ecchymosis, much of which may have arisen as a consequence of the repeated paroxysms and the obstructed respiration. The same may be said of the appearances in the spinal cord, which are generally very insignificant, or are open to much doubt; nor do our researches in the ganglia and the nerves carry us any further. The vascular appearances which have been seen in the fauces, the pharynx, the œsophagus, and the stomach, are by no means constant, though they often exist; and in one case which I saw examined, the attachment of the whole lining membrane of the œsophagus to the subjacent tissue was in a remarkable way weakened, so that the membrane was drawn out with the greatest facility like the finger of a glove; and I have heard the same condition described in another case. All these appearances, however, vary so much, that they must be considered the evidence of effects rather than of causes. The most marked deviation from health is usually found in the lungs, where great congestion is often discovered, and the lining membrane of the trachea and bronchial tubes is of a completely chocolate colour from the same cause. The obstruction to the passage of the air is often so great, or the sudden exertion in respiration so strong, that it is by no means uncommon to find that a partial emphysema has taken place, and that the air is thrown out into the cellular membrane which connects the different lobules of the lungs;—but this is only to be considered a result, and is occasionally found in other convulsive diseases, as in Epilepsy." 608.

Although, therefore, we have hitherto failed to discover any *anchora sacra* for this disease, the fact of its appearing both by its symptoms and pathology to be wholly functional, not only proves its remediability, but encourages the hope that a more adequate acquaintance with the structure and functions, nature and habitudes of the nervous system may divest our present treatment of hydrophobia, as well as of several other equally obscure though less fatal maladies, of empiricism, by teaching us how its functions have been deranged, by what means they may be re-adjusted, and to what extent their derangement is subject to remedy.

With this disease Dr. Bright closes his *third section* on Irritation, and with this section do his observations on the diseases of the brain and nervous system conclude. Twenty-five *additional cases,* illustrative of various points in both volumes, and *a concise statement of the diseased appearances of the brain and its membranes,* succeed to the paragraph on hydrophobia; but, although valuable in themselves, it is unnecessary for us to refer to these *addenda* more explicitly, than by observing that the Doctor's recapitulatory description of the morbid anatomy of the nervous system is the most concise, methodical, accurate and minute, which we have ever read. Regarding it as a condensed and generalized dissection of the 310 cases, with which these *Reports* are enriched, its value can only be estimated by the practical pathologist, who alone can judge of the labor, time, industry and talents which were necessary to collect the *materiel* out of which such a pathological epitome was constructed. To this is subjoined a series of plates illustrative of all the principal diseased appearances of the brain and meninges, and were any thing required to render these reports complete it was just such a collection of engravings. To forty splendidly-coloured drawings a most faithful and scientific pencil has transferred the morbid and multiform appearances, which the dissector's scalpel has disclosed; and in whatever light these plates are viewed, they stand unequalled by any which the English school has yet produced, while we know of none upon the Continent with which they may not confidently compete. The Doctor's first volume of Reports was accompanied by drawings, which perhaps occasionally displayed the painter's skill as strongly as the precise fac-simile of the actual disease, but in these before us the coloring is seldom more vivid than the original may be imagined to have been, and wherever the appearances to be depicted were not of the very deepest tinge, in two or three instances of which (*see* plates 17 and 31) the pencil was somewhat overcharged, nothing can be more precise, scarcely excepting the original. We would more especially refer to the 1st, 5th, 6th, 12th, 24th, and 30th plates, in confirmation of this opinion; but, although these are evidently the most happily executed, they all are highly creditable both to the author and the artist.

We cannot help regretting that a work of such truly practical importance as the present must be excluded by its price from very many of our libraries, and our regret is the greater when we find that this disqualifying property consists in the costliness and number of the drawings by which its other contents are at once so ornamented and enhanced. Sorry should we be to discourage the progress of an art, which has only begun to shew how auxiliary it may prove to the advancement of anatomical and pathological knowledge. But, however conducive faithful and vivid drawings must be to the full illustration and accurate comprehension of morbid results, which no words can adequately delineate, still it cannot be denied that solid, practical and scientific facts are of infinitely more value, and should neither be sacrificed nor suppresed for the sake of ornamental embellishments. The fringe, unquestionably, adds both to the warmth and worth of the garment, and where it can be had without seriously affecting the price of the article, or tending to limit the extent of its usefulness, it may be purchased with advantage. But when its costliness is so extreme as to treble its selling price, and to place a useful article of wear beyond the reach of many who equally require and desire it, prudence would dictate to the seller as well as to the purchaser, the propriety of divesting the garment of its fringe entirely, or of ornamenting it with a border less expensive in its texture. Until the draughtsman can execute his task on more moderate terms, or until, as in most of the medical schools upon the Continent, our youth are taught to regard the pencil and box of colours equally essential parts of their instrumental apparatus with the forceps or the lancet, it would be our advice to medical writers to separate their text and plates, when the latter are so expensive or so numerous as materially to augment the selling value of the former.

When we lament that Dr. Bright has not pursued this course, we have more regard to the reader's interest than his; and in pressing upon the Doctor the adoption of the plan now recommended, when a second edition shall appear, it is much more from a desire to see the valuable facts and cases which these Reports contain circulating universally throughout the profession, than to suggest to the publisher a more convenient arrangement than the one adopted. Were these Reports printed in an octavo form, with a small type, and accompanied with their present plates in a distinct volume, ten copies would be read for every copy which will now be disposed of, and the general practitioner, for whom they are so especially adapted, would not be forced to depend upon circulating libraries for any knowledge which he may obtain of their contents.

Few enjoy such opportunities for undisturbed and enlightened observation as Dr. Bright—few can test more fully every exploded doctrine and every novel remedy, and few are qualified to relate more dispassionately, clearly, and honestly the observations which they make. The two volumes, which we now close, are little inferior to an extensive hospital appropriated to diseases of the nervous system. They are quite equal to it in the number of their cases; they are not inferior to it in their variety; they are much superior to it in the well-digested histories with which these cases are accompanied; and, if we refer to the scientific inferences, practical rules and clinical philosophy with which they abound, no comparison can be instituted between them. It is unnecessary to say that a hospital in type is no common spectacle, and it were superfluous to add that one, the wards of which are ever open, and the beds of which are ever occupied, cannot be made to circulate too extensively. Many are no longer able to attend on hospital instruction; many are no longer willing; and not a few there are who, though both able and desirous, are restrained by feelings which it is unnecessary to specify. To each of these the work before us is equally recommended; for he who is either unable or unwilling to come to clinical instruction, may be induced to study it if clinical instruction come to him; and as the first ticket procures him permanently free admission, without the favour of lecturers, or the certificate of governors, he can neither value his own improvement, nor that of the science to which he officially belongs, if he neglect to purchase.

